# Cancer therapy by cyclin-dependent kinase inhibitors (CDKIs): bench to bedside

**DOI:** 10.17179/excli2024-7076

**Published:** 2024-06-04

**Authors:** Ali Hassanzadeh, Navid Shomali, Amin Kamrani, Mohammad Sadegh Soltani-Zangbar, Hadi Nasiri, Morteza Akbari

**Affiliations:** 1 Department of Applied Cell Sciences, School of Advanced Technologies in Medicine, Tehran University of Medical Sciences, Tehran, Iran; 2 Department of Immunology, Faculty of Medicine, Tabriz University of Medical Sciences, Tabriz, Iran; 3 Immunology Research Center, Tabriz University of Medical Sciences, Tabriz, Iran; 4 Department of Medical Biotechnology, Faculty of Advanced Medical Sciences, Tabriz University of Medical Sciences, Tabriz, Iran

**Keywords:** cancer, cyclin-dependent kinases (CDKs), CDK inhibitors (CDKIs), treatment

## Abstract

A major characteristic of cancer is dysregulated cell division, which results in aberrant growth of cells. Consequently, medicinal targets that prevent cell division would be useful in the fight against cancer. The primary regulator of proliferation is a complex consisting of cyclin and cyclin-dependent kinases (CDKs). The FDA has granted approval for CDK inhibitors (CDKIs) to treat metastatic hormone receptor-positive breast cancer. Specifically, CDK4/6 CDKIs block the enzyme activity of CDK4 and CDK6. Unfortunately, the majority of first-generation CDK inhibitors, also known as pan-CDK inhibitors because they target multiple CDKs, have not been authorized for clinical use owing to their serious side effects and lack of selection. In contrast to this, significant advancements have been created to permit the use of pan-CDK inhibitors in therapeutic settings. Notably, the toxicity and negative consequences of pan-CDK inhibitors have been lessened in recent years thanks to the emergence of combination therapy tactics. Therefore, pan-CDK inhibitors have renewed promise for clinical use when used in a combination regimen. The members of the CDK family have been reviewed and their primary roles in cell cycle regulation were covered in this review. Next, we provided an overview of the state of studies on CDK inhibitors.

## Introduction

The highly controlled procedure referred to as the cell cycle allows for the development, genetic material duplication, and dividing of cells (Zhang et al., 2019[132]; Zhou, 2017[135]). The core cell-cycle apparatus functioning in the cell nucleus is what propels the development of the cell cycle from one phase to the next. The proteins known as cyclins and their catalytic partners, the cyclin-dependent kinases (CDKs), make up this apparatus. During distinct stages of the cell cycle, distinct cyclin-CDK complexes trigger and phosphorylate their target proteins. The transcription specific to each cell cycle, protein breakdown, and a number of CDK-inhibitor proteins all work together to closely control the function of cell-cycle proteins in normal cells (Whittaker et al., 2017[116]). All these processes tend to get dysregulated in tumors in humans, leading to abnormal stimulation of cell-cycle proteins. In fact, the majority of kinds of tumors grow as a consequence of genetic lesions that cause the core cell-cycle machinery to become hyperactivated (M Manohar, 2022[75]; Sofi et al., 2022[106]). For these explanations, targeting cell-cycle proteins appear to indicate a successful means of stopping tumor growth. The initial excitement around blocking cyclin-CDK kinases was tempered by the general consensus that these kinds of proteins are necessary for the growth of normal, non-transformed cells (Roskoski, 2019[99]). Nevertheless, individual CDKs and cyclins are essentially unnecessary for the growth of normal tissues, according to genetic experiments. On the other hand, based on the genetic abnormalities they carry, such proteins are necessary for the growth of particular kinds of tumors. CDK2 is believed to regulate the transition into the S phase of the cell cycle, whereas CDK1 governs the commencement of mitosis. Recent research indicates that CDK1 may effectively facilitate the G1/S transition in CDK2−/− cells. This raises the issue of whether CDK1 is the primary cyclin-dependent kinase in mammalian cells or whether it just compensates for the absence of CDK2 (Bashir and Pagano, 2005[5]). Function of CDK1 and CDK2 in animal models of cancers has been summarized in Tables 1(Tab. 1) and 2(Tab. 2). Inhibitors of CDK4 and CDK6 are the most blatant examples of cell-cycle machinery targeting achievement (Panagiotou et al., 2022[89]). The implementation of these compounds into clinical practice marked a significant advancement in the management of breast cancer and is expected to have a significant impact on the management of numerous different kinds of cancer as well (Malumbres and Barbacid, 2009[78]). Surprisingly, new research has shown that cell-cycle proteins influence the microenvironment of tumors as well as the cancer cells themselves, possibly through modifying the immune system's response against the tumor (Javed et al., 2023[55]; Malumbres, 2014[77]). Therefore, it is probable that blocking such proteins will have an impact on various facets of the carcinogenic process. Even with the present effectiveness of CDK4/6 inhibitors (CDK4/6i), the field of cancer therapy remains in its infancy when it comes to targeting cell-cycle proteins (García-Reyes et al., 2018[40]; Gupta et al., 2023[45]). In this review, we address the present situation and offer our recommendations for the most announcing course of action.

## Key Checkpoints in the Cell Cycle and Their Regulatory Mechanisms

The cell cycle undergoes meticulous regulation via checkpoints, primarily the G1, G2, and M checkpoints.

### G1 checkpoint

Also known as the restriction point, the G1 checkpoint, positioned at the G1 phase's conclusion, monitors external signals (growth factors, nutrient availability) and internal signals (DNA integrity, cell size). Regulation involves cyclin-dependent kinases (CDKs), cyclins, and tumor suppressor protein p53. CDK-cyclin complexes facilitate progression, while p53 addresses DNA damage, triggering repair or apoptosis (Gao et al., 2020[39]).

### G2 checkpoint

Situated at the G2 phase's conclusion, the G2 checkpoint ensures DNA replication completion and repair before mitosis. CDK-cyclin complexes, notably CDK1-cyclin B, govern this checkpoint. Checkpoint kinases (CHK1 and CHK2) respond to DNA damage, halting the cell cycle for repair (Ding et al., 2020[32]).

### M checkpoint

Also termed the spindle checkpoint, the M checkpoint operates during mitosis, confirming accurate chromosome segregation. Monitoring chromosome attachment to the mitotic spindle, this checkpoint relies on tension-sensing at kinetochores. Proteins such as BUB1, BUBR1, and MAD2 are pivotal, inhibiting anaphase-promoting complex/cyclosome (APC/C) until proper attachment and tension are achieved (Chou et al., 2020[21]).

Collectively, these checkpoints are integral in preserving genomic integrity, preventing damaged DNA propagation, and ensuring cell cycle progression under suitable conditions. Dysregulation of these checkpoints can lead to uncontrolled cell growth, genomic instability, and diseases, including cancer (Asghar et al., 2015[2]).

## G1 Checkpoint and its Role in Cell Growth and DNA Damage Repair

The G1 checkpoint, or restriction point, represents a crucial regulatory juncture occurring at the G1 phase's conclusion before entering the S phase. This checkpoint orchestrates cell growth, assesses environmental signals, and ensures DNA integrity before committing to replication and division. Functioning as a decision node, the G1 checkpoint determines whether the cell proceeds with the cycle, temporarily enters G0, or undergoes apoptosis (Smith et al., 2020[105]). It scrutinizes various signals, both internal and external, ensuring favorable conditions for division. Integral to the G1 checkpoint is its monitoring of cell size, ensuring sufficient growth before DNA replication. Additionally, the checkpoint assesses DNA damage, activating p53-mediated signaling pathways for DNA repair, cell cycle arrest, or apoptosis based on damage severity (Saleem et al., 2018[100]). In essence, the G1 checkpoint is a pivotal control point governing cell growth, monitoring DNA integrity, and coordinating decisions regarding replication and division. Dysfunction or loss of this checkpoint can lead to unbridled cell proliferation, genomic instability, and disease, including cancer (Barnaba and LaRocque, 2021[4]).

## G2 Checkpoint and its Role in DNA Replication Fidelity

The G2 checkpoint, a pre-mitotic regulatory juncture, operates at the G2 phase's culmination, ensuring the accuracy of DNA replication before mitosis. This checkpoint acts as a quality control mechanism, verifying precise DNA replication and effective repair of any incurred damage (Pedroza-Garcia et al., 2022[92]). Central to the G2 checkpoint is the cyclin-dependent kinase 1 (CDK1) in complex with cyclin B, which plays a key role in the transition from G2 to mitosis. This activation is crucial for mitotic entry. The checkpoint also involves checkpoint kinases (CHK1 and CHK2), activated in response to DNA damage, halting the cell cycle for repair. The G2 checkpoint, coordinating with the DNA damage response pathway involving p53, provides an opportunity for cells to repair DNA damage before mitosis (Dillon et al., 2014[31]). This ensures replication fidelity, prevents genetic abnormalities' transmission, and upholds genomic stability. In summary, the G2 checkpoint is pivotal in maintaining DNA replication accuracy, relying on CDK1-cyclin B, checkpoint kinases, and p53-mediated signaling pathways. Dysregulation or loss of this checkpoint can lead to genomic instability and the accumulation of DNA damage, contributing to disease, including cancer (Barnaba and LaRocque, 2021[4]).

## M Checkpoint and its Role in Ensuring Accurate Chromosome Segregation

The M checkpoint, known as the spindle checkpoint, holds critical regulatory significance during mitosis. Its primary function is to ensure the precise segregation of chromosomes into daughter cells. As duplicated chromosomes condense and align at the metaphase plate during mitosis, the M checkpoint monitors chromosome attachment to the mitotic spindle. This is achieved through tension-sensing at kinetochores, protein complexes linking chromosomes to spindle microtubules (Pedroza-Garcia et al., 2022[92]; Uzbekov and Prigent, 2022[113]). The M checkpoint employs a signaling pathway involving checkpoint kinases (BUB1, BUBR1, MAD2) at kinetochores of unattached or misaligned chromosomes. In the absence of proper attachment or tension, these proteins inhibit the anaphase-promoting complex/cyclosome (APC/C), delaying anaphase until correct attachment and tension are achieved. The M checkpoint's role is pivotal in ensuring each daughter cell receives the correct chromosome number, preventing aneuploidy and genomic instability (Dillon et al., 2014[31]). Proper M checkpoint regulation is essential to avoid chromosome missegregation, safeguarding against diseases such as cancer (Curry and Lim, 2015[28]).

## Dysregulation of Cell Cycle Control in Cancer

Dysregulation of cell cycle control is a fundamental characteristic of cancer, where the meticulously regulated mechanisms governing normal cell cycle progression and genomic stability are disrupted. This dysregulation manifests as uncontrolled cell proliferation, genomic instability, and the initiation of tumor formation. The following factors contribute significantly to the dysregulation of cell cycle control in cancer:

### Mutations in cell cycle regulatory genes

Genetic mutations in cell cycle regulatory genes, such as tumor suppressor genes like p53, retinoblastoma protein (Rb), and cyclin-dependent kinase inhibitors (CDKIs), can impair their normal functions. These mutations result in uncontrolled cell cycle progression as the regulatory mechanisms are compromised (Cordon-Cardo, 1995[26]; Liggett and Sidransky, 1998[71]; Yadav et al., 2018[124]).

### Overactivation of oncogenes

Oncogenes, responsible for promoting cell growth and division, can be excessively activated in cancer cells. This activation, achieved through gene mutations or amplification, leads to heightened production or activity of proteins involved in driving cell cycle progression. Examples include cyclins and cyclin-dependent kinases (CDKs), which, when mutated or amplified, contribute to uncontrolled cell cycle progression (Chen et al., 2022[17]; Link, 2019[72]).

### Loss of checkpoint control

Dysregulation or loss of checkpoints in the cell cycle, such as the G1, G2, and M checkpoints, allows cells with damaged DNA or chromosomal abnormalities to continue dividing. Failure to properly arrest the cell cycle at these checkpoints facilitates the propagation of cells with genomic alterations, thereby contributing to the development of tumors (Engeland, 2018[35]; Mens and Ghanbari, 2018[79]).

### Disrupted DNA damage response

Cancer cells often exhibit defects in DNA repair mechanisms, resulting in the accumulation of DNA damage. Compromised DNA repair mechanisms in cancer cells allow damaged DNA to persist, triggering checkpoint activation and cell cycle arrest. However, in cancer cells, these mechanisms are compromised, enabling cells with damaged DNA to continue dividing and acquiring additional mutations (Johnson et al., 2009[57]; Lee et al., 2021[68]; Li et al., 2022[69]).

### Abnormal expression of cell cycle regulators

Altered expression levels or activity of cell cycle regulators, including cyclins, CDKs, and associated proteins, disrupt the delicate balance of cell cycle progression. Overexpression of cyclins or constitutive activation of CDKs can drive cells through the cell cycle even in the absence of appropriate signals (Schafer, 1998[102]). The dysregulation of cell cycle control in cancer results in unbridled cell proliferation, evasion of growth suppressors, and the ability to bypass normal checkpoints. This uncontrolled cell division contributes significantly to the formation and progression of tumors (Kamranvar et al., 2022[59]). A comprehensive understanding of the specific molecular alterations in cell cycle control in various cancer types offers valuable insights for the development of targeted therapies aimed at restoring normal cell cycle regulation and inhibiting cancer cell growth.

## Mutations and Alterations in Cell Cycle Regulators

Mutations and alterations in cell cycle regulators wield profound influence over cell cycle control and significantly contribute to cancer development. An illustrative instance is the dysregulation of cyclins and CDKs, pivotal in steering cell cycle progression by phosphorylating target proteins (Wenzel and Singh, 2018[115]). Mutations or alterations in these regulators can disrupt the meticulous orchestration of the cell cycle. For instance, the overexpression of cyclin D1, observed in various cancers, propels uncontrolled cell proliferation by activating CDK4/6. Tumor suppressor genes, instrumental in cell cycle regulation, are susceptible to mutations, impacting normal cell growth. TP53, a prominent tumor suppressor gene encoding the p53 protein, frequently undergoes mutation in cancer (Bonn et al., 2012[8]; Leake, 1996[67]). Loss of p53 function compromises its ability to regulate the G1 checkpoint, DNA repair, and apoptosis. The retinoblastoma protein (Rb) serves as another crucial cell cycle regulator, governing the G1/S transition by inhibiting E2F transcription factors. Mutations in the RB1 gene or alterations in the Rb protein can disrupt cell cycle control, as evident in retinoblastoma and osteosarcoma. Checkpoint proteins such as CHK1, CHK2, and MAD2 play pivotal roles in surveilling DNA integrity, ensuring precise cell cycle progression. Mutations in these checkpoint proteins can impede their ability to detect DNA damage or defects in chromosome alignment, resulting in genomic instability and an augmented cancer risk. These mutations and alterations in cell cycle regulators disrupt the precise control mechanisms dictating cell division and DNA integrity (Bao and Hua, 2014[3]). Consequently, uncontrolled cell proliferation, genomic instability, and the accumulation of genetic alterations occur, contributing to the development and progression of cancer. A nuanced comprehension of these alterations in specific cell cycle regulators provides critical insights into the underlying mechanisms of cancer and informs the development of targeted therapies aiming to restore normal cell cycle control and impede tumor growth.

## CDK4/6 Inhibitors

Produced in 2001, palbociclib became the initial CDK4/6-specific inhibitor to demonstrate activity versus a variety of human cancer cell lines and xenografts, including breast tumors. CDK4/6 inhibition was most effective in suppressing breast cancer cell lines that represented hormone receptor-positive (HR+), luminal-type mammary carcinomas. The use of CDK4/6i (palbociclib, ribociclib, and abemaciclib) in clinical studies with humans with breast cancer resulted from this data as well as the animal genetic studies previously mentioned (Pandey et al., 2019[90]; Yang et al., 2020[125]). The initial clinical trial began in 2007 and proved palbociclib's efficiency versus mantle cell lymphoma (Spurgeon et al., 2017[107]). Phase II and III clinical studies including palbociclib (PALOMA series), ribociclib (MONALEESA), and abemaciclib (MONARCH) were conducted since 2015 (Mo et al., 2022[80]; O'Leary et al., 2016[86]). In patients with advanced HR+/HER2− breast tumors, these studies examined the efficacy of combination CDK4/6i with standard endocrine therapy (an aromatase inhibitor, letrozole, or an estrogen receptor antagonist, fulvestrant). The incorporation of any of these CDK4/6i significantly increased patients' survival rates and progression-free survival. Additionally, once administered as monotherapy, abemaciclib increased PFS l in females with HR+/HER2− metastatic breast cancer (Goel et al., 2017[41]; Goyal et al., 2023[43]). As a result, the US Food and Drug Administration (FDA) authorized the use of all three CDK4/6i drugs in the management of patients who have advanced or metastatic HR+/HER2− breast cancer.

Although abemaciclib additionally blocks a number of other kinases, palbociclib and ribociclib are highly selective inhibitors of CDK4 and CDK6, respectively (Zeverijn et al., 2023[129]; Zhu and Zhu, 2023[136]). For palbociclib and ribociclib, neutropenia, thrombocytopenia and anemia constitute the restricting toxicity. Because hematopoietic cells suppress cyclin D3-CDK6, this action is probably on goal (Kwapisz, 2017[65]; Petrelli et al., 2019[93]). Conversely, these adverse reactions tend to be less prominent in patients treated with abemaciclib, which could be attributed to the increased effectiveness of this chemical in suppressing CDK4 instead of CDK6 (Groenland et al., 2020[44]). Because of this, abemaciclib doesn't need an intermittent dosage schedule, unlike palbociclib and ribociclib. Gastrointestinal adverse reactions are most common among patients using abemaciclib (Cameron et al., 2023[13]; Cejuela et al., 2023[15]; Colombo et al., 2023[25]; Groenland et al., 2020[44]). Although the chemical mechanism is unidentified it was proposed that the blocking of CDK9 by abemaciclib might be the cause of this event.

Whether abemaciclib's capacity to block additional kinases is a benefit or a drawback compared to CDK4/6i, which is more selective, is still up for debate (Braal et al., 2021[12]). According to a new PALLAS research, palbociclib did not increase metastatic disease-free survival when added to adjuvant endocrine therapy for individuals with early-stage HR+/HER2-breast cancer as compared with adjuvant endocrine therapy alone. On the other hand, a comparable trial with abemaciclib revealed a noteworthy extension of the time without invasive illness (Klein et al., 2018[61]). The medical benefit of abemaciclib might be attributed to its capacity to inhibit other kinases. It's also possible that abemaciclib's off-target impacts contribute to its effectiveness as a single drug.

Development of sensitivity and resistance biomarkers is an urgent need for CDK4/6i therapy. It is well known that when CDK4/6 is inhibited, tumor cells that no longer express RB1 do not stop proliferating. In fact, the strongest predictor of CDK4/6i effectiveness is intact RB1 state. Some predictors remain unclear. Cancers with genomic activation of CCND1-3 genes were observed to be especially vulnerable to CDK4/6i (Krasniqi et al., 2022[63]; Roberts et al., 2020[98]). Post hoc evaluations of the PALOMA trials revealed that responsiveness to CDK4/6i was not linked with either CCND1 amplification or cyclin D1 mRNA levels. Various investigations have linked CDK4 gene amplification and protein overexpression to greater sensitivity or resistance, although the predictive usefulness of such lesions is unknown (Fontanella et al., 2022[37]; Hsu et al., 2022[53]). It has been demonstrated that susceptibility to palbociclib treatment in breast cancer may be predicted by immunohistochemical identification of Thr172-phosphorylated CDK4 (an activating phosphorylation performed through the CDK-activating kinase, CAK). This finding might offer a prognostic indicator that can be evaluated in tumor tissues. Conversely, it has been demonstrated that resistance to palbociclib is correlated with elevated levels of cyclin E1 mRNA in metastatic lesions (Billard-Sandu et al., 2020[6]; Braal et al., 2021[12]). Even with the advancements, more markers are required to accurately forecast how patients will react to CDK4/6 inhibition. Function of CDK4/6 in animal models of cancer has been summarized in Table 3(Tab. 3).

## CDK7 Inhibitors

Due to its dual roles in transcriptional regulation and cell cycle control, CDK7 is a promising target for treatment of tumors. Substantial anti-tumor action has been demonstrated for a number of CDK7-specific inhibitors, such as the covalent inhibitors THZ1, THZ2, YKL-5-124, and non-covalent inhibitors BS-181, ICEC0942, LDC4297, and QS1189. BS-181 is an initial extremely specific CDK7 inhibitor (Zhang et al., 2020[130], 2021[133]). Although BS-181 has a low bioavailability and inadequate cell permeability, preclinical investigations have demonstrated that it suppresses the growth of tumor cells and the formation of xenograft tumors. The first oral CDK7 inhibitor, ICEC0942 (CT7001), was created from BS-181 and had more drug-like qualities than BS-181 (Sava et al., 2020[101]). Particularly, ICEC0942 submitted clinical trials in 2017 and is now being studied in phase I/II trials for an array of treatments for advanced cancers, involving monotherapy or combination therapy for triple-negative breast cancer, castrate resistant prostate cancer (CRPC), and combination therapy with Fulvestrant for patients with HR+/HER2- breast cancer (ClinicalTrials.gov identifier: NCT03363893) (Kovalová et al., 2023[62]; Kumar et al., 2021[64]; Panagiotou et al., 2022[89]; Petroni and Galluzzi, 2020[94]). One of the most extensively researched CDK7 covalent antagonists is THZ1. Significant anti-tumor efficacy of THZ1 has been demonstrated in preliminary research in a variety of tumor types (Kovalová et al., 2023[62]). Notably, it was demonstrated that THZ1 inhibits CDK12 and CDK13 functions in addition to CDK7 function. Scientists combined the pyrrolidinopyrazole core of PAK4 inhibitor PF-3758309 with the covalent warhead of THZ1 to create the antagonist YKL-5-124, which is a more selective CDK7 inhibitor (Sava et al., 2020[101]). While YKL-5-124 exhibits no inhibitory property towards CDK12 or CDK13, it shows a strong selectivity for blocking CDK7. Preclinical research has demonstrated that in small cell lung cancer, YKL-5-124 can enhance genomic instability and initiate an immune response against the tumor. This offers an empirical basis for combining the treatment of CDK7 antagonists with immunotherapy (Sava et al., 2020[101]). In May 2017, a phase I clinical trial including advanced solid tumors was launched to assess the effectiveness of SY-1365, a CDK7 inhibitor derived from THZ1, in treating breast and ovarian cancer (Clopper and Taatjes, 2022[24]; Diab et al., 2020[30]). (ClinicalTrials.gov identifier: NCT03134638). SY-5609 is another selective CDK7 inhibitor and preclinical testing has demonstrated that SY-5609 and Fulvestrant together exhibit strong anti-cancer action against ER+ breast cancer, TNBC, and ovarian cancer (Diab et al., 2020[30]; Kovalová et al., 2023[62]). SY-5609 commenced phase I clinical studies for the management of advanced solid tumors and in conjunction with Fulvestrant for women with HR+/HER2- breast cancer (ClinicalTrials.gov identification: NCT04247126).

## CDK9 Inhibitors

Since it controls cellular transcriptional elongation and mRNA maturing, CDK9 has gained attention as an intriguing therapy for a variety of malignancies, particularly those brought on by transcriptional deregulation. Numerous CDK9 inhibitors, including Fadraciclib, AZD-4573, CDKI-73, MC180295, and others, were discovered, and preliminary researches have revealed their considerable anti-cancer potential (Wu et al., 2023[120]; Xie et al., 2020[122]; Yao et al., 2022[126]). Additionally, permanent suppression of MYCN-amplified neuroblastoma may be achieved with the combination of temozolomide and facraclib. A highly targeted CDK9 inhibitor, AZD-4573, has the ability to suppress the expression of MCL-1 and other carcinogenic genes. AZD-4573 is a highly effective tumor-fighting treatment for blood cancers (Wu et al., 2020[118][119], 2023[120]). Olaparib and CDKI-73 work synergistically to treat BRCA1-positive ovarian cancer, which makes it easier to employ CDK9 as a predictive biomarker for PARP antagonists in clinical trials (Morillo et al., 2023[83]). Tumor suppressor gene expression can be restored by MC180295 by dephosphorylating the SWI/SNF protein Brg1, promoting gene activation. Furthermore, CDK9 reduction makes a good target for epigenetic treatments against malignancy since it sensitizes to the immune checkpoint inhibitor α-PD-1 *in vivo* (Cidado et al., 2020[23]; Freeman-Cook et al., 2021[38]; Hu et al., 2023[54]; Karati et al., 2023[60]). The development of CDK9 inhibitors for clinical use has been aided by the findings from these animal investigations. Because of their severe side effects and low selectivity, four CDK9 inhibitors, P276-00, ZK-304709, BAY-1000394, and SNS-032, have had their clinical trials halted (Borowczak et al., 2020[10], 2022[9]; Cidado et al., 2020[23]; Freeman-Cook et al., 2021[38]). 

## CDK12 Inhibitors

In addition to CDK7 and CDK9, CDK12 is a crucial transcriptional regulator within the CDK family. It has the ability to attach to cyclin K and phosphorylate RNA polymerase II's CTD region, which encourages transcription extension (Rebuzzi et al., 2022[97]; Wu et al., 2018[121]; Zhang et al., 2016[134]). New studies have discovered several unique activities of CDK12 in cancer, particularly breast cancer. Numerous biological processes, such as c-MYC expression, Wnt/β-catenin signaling, RNA splicing, ErbB-PI3K-AKT signaling, MAPK signaling, noncanonical NF-kB pathway, and DNA damage response (DDR) signaling, are regulated in order to accomplish such unique roles (Maity et al., 2023[76]; Mounika et al., 2023[84]; Niu et al., 2022[85]; Quereda et al., 2019[95]). THZ531 and SR-4835 are two CDK12 inhibitors that showed significant anti-tumor effectiveness in preliminary research. SR-4835 is a very specific dual inhibitor of CDK12 and CDK13 that has the ability to block the expression of key proteins involved in DNA damage response (Cesari et al., 2023[16]; Cheng et al., 2022[20]). In TNBC, this may incite a "Brcaness" phenotype that results in impairments in DDR, hence enhancing the combined effect of PARP antagonists and DNA damage treatment (Emadi et al., 2020[34]). Another covalent inhibitor of CDK12 and CDK13, THZ531, has the ability to dramatically suppress the expression of genes involved in the DDR as well as important transcription factors connected to super-enhancers (He et al., 2022[46]; Liu et al., 2021[73]). According to recent research, THZ531 and sorafenib work remarkably well together to treat HCC. Dinaciclib is one of the pan-CDK inhibitors that has been used to target CDK12 in clinical trials thus far (Criscitiello et al., 2014[27]; Howard et al., 2021[52]; Raina et al., 2023[96]). Thus, it is necessary to design CDK12 antagonists with excellent specificity and medicinal qualities.

## Pan-CDK Inhibitors

Since the 1990s, inhibitors of the CDK have been researched. The pan-CDK inhibitors, such as roscovitine and flavopiridol, are among the first class of CDK inhibitors. Through reducing the functioning of the CDK enzyme, such inhibitors primarily prevent the cell cycle and hinder cell division. Nevertheless, the first-generation pan-CDK inhibitors are very toxic and display limited selectivity, which inevitably causes negative effects on normal cells. Thus, the majority of pan-CDK inhibitors were found to be ineffective in their clinical trials. More selective as well as fewer opposite effect-prone second-generation CDK inhibitors have since been established, such as RGB-286638, AT7519, TG02, Dinaciclib, P276-00, and so forth (Stone et al., 2012[108]; Xie et al., 2022[123]). The majority of second-generation inhibitors of CDK have demonstrated effective anti-cancer effects in preclinical trials; however, additional clinical studies are required to confirm the safety and efficacy of such inhibitors. Currently, about forty pan-CDK inhibitors are being investigated and developed at different phases. For example, the Merck company's Dinaciclib is currently undergoing phase II clinical trials and has demonstrated a notable anti-cancer impact in the management of leukemia, breast cancer, and melanoma (Heptinstall et al., 2018[47]; Panagiotou et al., 2022[89]). In addition, a number of pan-CDK inhibitors have been the subject of phase I or phase II studies, and preclinical research has demonstrated a strong anti-cancer effect for numerous other pan-CDK inhibitors. Several investigations have been done on drug delivery strategies, particularly in the field of combination therapy, to reduce the adverse reactions of pan-CDK inhibitors. Pan-CDK inhibitors have generally demonstrated encouraging clinical efficacy, despite severe adverse effects and safety issues (Chen et al., 2012[18]; Dukelow et al., 2015[33]; Jhaveri et al., 2021[56]). Here, we have enumerated pan-CDK inhibitors that are presently being investigated and developed, along with a summary of their structures, developmental stages, CDK targets, and signs of target illnesses or malignancies. The detailed description of representative pan-CDK inhibitors is provided below.

### Flavopiridol

Alvocidib, also known as flavopiridol, is a member of the first pan-CDK inhibitor generation. It has been one of the most extensively researched pan-CDK inhibitors and the first to be used in clinical trials. The primary activities of CDK1, CDK2, CDK4, CDK6, CDK7, and CDK9 are inhibited by flavopiridol (Tan and Swain, 2002[109]). Flavopiridol, which is primarily administered for the treatment of ALL, AML, CLL, lymphomas, solid tumors, gastric cancer, mantle cell lymphomas, myeloid leukemia, and other conditions, has been the subject of 63 clinical trials since 1997. According to preclinical research findings, flavopiridol demonstrated strong anti-tumor effects toward prostate cancer, 85 % tumor volume reduction and 30-day survival extension (Tong et al., 2023[111]; Wang and Ren, 2010[114]). Furthermore, *in vitro*, flavopiridol can cause primary and recurrent/refractory AML cells to undergo death by a factor of 4.3 (Christian et al., 2007[22]; Hosono, 2019[49]; Joshi et al., 2023[58]). Numerous other hematopoietic cell lines are also susceptible to apoptosis induction by it. Despite these encouraging preclinical study developments, flavopiridol demonstrated subpar efficacy in solid tumor clinical trials. After treatment with flavopiridol, the total amount of peripheral blood cells dropped by more than 50 % in 44 % of patients, according to phase I clinical studies of AML (Zeidner and Karp, 2015[128]). This suggests that flavopiridol may cause anti-leukemia cytotoxicity. Following this, a phase II clinical research was conducted on 45 AML patients, and throughout the course of therapy, 16 % of the patients experienced cardiac failure. Clinical trials of CLL patients in Phase I and Phase II have demonstrated that flavopiridol can reduce symptoms (Wiernik, 2016[117]). Flavopiridol's adverse effects hindered the advancement of clinical trials. To increase Flavopiridol's clinical efficacy and lessen its side effects, scientists are attempting to mix it with other cancer treatments (Bozok Cetintas et al., 2016[11]; Hicks et al., 2014[48]). Selected overview of advanced clinical trials of Alvocidib has been provided in Table 4(Tab. 4).

### Dinaciclib

Merck & Co. Ltd.'s dinaciclib (SCH727965) has reached phase III clinical trials and demonstrated remarkable anti-cancer effectiveness against breast cancer, lung cancer, and chronic lymphocytic carcinoma. Dinaciclib primarily inhibits CDK9 action, which stops phosphorylation of RNA polymerase II's carboxyl terminus (Schott et al., 2024[103]; Teng et al., 2023[110]; Tsao et al., 2022[112]). Phosphorylation of this terminus slows transcription and causes cell death. Remarkably, research has shown that Dinaciclib is the most effective treatment for leukemia. Dinaciclib extended the survival time of mouse cancer xenograft models and reduced the development of T-ALL cells in acute lymphoblastic leukemia (Teng et al., 2023[110]; Yun et al., 2019[127]). Dinaciclib and Panobinostat together have been shown in preclinical studies to cause MLL-AF9 cancer cell death. The median survival rose in the mouse tumor model, indicating a larger survival benefit due to the considerable reduction of leukocytes. Subsequent research revealed that Dinaciclib is capable of removing a wide range of cytokines from the microenvironment, including those necessary for the proliferation of CLL cells including CD40L, BAFF, IL-4, and others (Moharram et al., 2017[81]; Pariury et al., 2023[91]). According to such researches, dinaciclib offers a lot of promise for use as a therapeutic treatment drug for CLL (Chen et al., 2016[19]; Fabre et al., 2014[36]). The outcomes of clinical trials also demonstrated that Dinaciclib was more effective for CLL than Flavopiridol. Recent research has also shown that dinaciclib, in combination with PD1 monoclonal antibodies, has an even more strong anti-cancer impact, indicating that dinaciclib may be a very interesting therapeutic target in a clinical environment (Hossain et al., 2018[50]; Howard et al., 2022[51]; Li et al., 2024[70]; Pariury et al., 2023[91]).

### P276-00

P276-00 shows great capacity in inhibiting CDK1, CDK4, and CDK9 in MCL cells (Cassaday et al., 2015[14]; Shirsath et al., 2012[104]). Thirteen patients with relapsed and refractory MCL were treated with p276-00 in the Phase II clinical trial of MCL. In general, there was a considerable anti-tumor impact as well as medication resistance. The exact chemical mechanism by which p276-00 treats MCL is still unknown (Cassaday et al., 2015[14]; Shirsath et al., 2012[104]). According to other research, p276-00 can cause head and neck cancer cells to undergo death by stopping the cell cycle in the G1 phase. Patients with recurrent and locally advanced head and neck cancer participated in a phase II clinical trial to assess the anti-cancer effects and safety of p276-00 (Cassaday et al., 2015[14]; Shirsath et al., 2012[104]). P276-00 appeared to have excellent anti-cancer effectiveness based on the findings; nevertheless, more research is required to determine its safety.

### TG02

A brand-new oral poly-kinase inhibitor called TG02 primarily blocks the actions of CDK1, CDK2, CDK7, and CDK9. Preclinical research has demonstrated that TG02, either by itself or in conjunction with TMZ, can stop glioblastoma cells from proliferating. Scientists have carried out phase I clinical trials to ascertain the clinical dosage and effectiveness of TG02 (Le Rhun et al., 2023[66]; Lohmann et al., 2020[74]). The outcomes demonstrated the efficacy of TG02 in the management of hematological malignancies, and it was discovered that TG02 therapy increases tumor formation and extends longevity in a range of leukemia mice models (Goh et al., 2012[42]; Pallis et al., 2012[87], 2017[88]). With its broad-spectrum anti-CDKs and anti-JAK2/Flt3 activities, TG02 offers an empirical basis for the therapeutic management of blood cancers (Goh et al., 2012[42]). Subsequent research has demonstrated that the combination of TG02 and carfilzomib, a second-generation proteasome inhibitor, enhances the effectiveness of relapsed or resistant multiple myeloma (MM) (Aleem and Arceci, 2015[1]; Blachly et al., 2016[7]). To sum up, TG02 has demonstrated encouraging therapeutic promise in clinical studies, but more research is still required in the future.

## Combination Therapy of CDK Inhibitors and PD1-PDL1 Antibodies

After years of study, cancer immunotherapy has become a potent and successful cancer therapy method. Dr. Honjo discovered PD1 (programmed death receptor 1) and showed that T cells expressed it in 1992. PDL1 (B7-H1) was discovered by Dr. Chen in 1999, and he also showed that immune and tumor cells express PDL1 highly (Moore et al., 2022[82]). The link between PDL1 and PD1 promotes T cell death and adversely impacts the stimulation of lymphocytes. As a result, inhibiting PD1-PDL1 immunological checkpoints encourages T cell activation, which makes it easier for T cells to kill cancer cells. Despite the extraordinary effectiveness of blocking the immune checkpoint PD1-PDL1 in the curative therapy of several tumors, the majority of cancer patients were unable to react satisfactorily to immunotherapy (Mounika et al., 2023[84]). Furthermore, with the targeted therapy of PD1-PDL1, drug resistance may develop. As a result, numerous studies are being carried out to enhance cancer patients' receptivity to immunotherapy by using combination therapy approaches. Certain CDK inhibitors have been found in recent research to strengthen the immune system's defense against tumors (Mounika et al., 2023[84]). Certain CDK inhibitors have shown strong anti-tumor effectiveness in preclinical and clinical trials if combined with PD1-PDL1 immunotherapy. One CDK4/6 inhibitor that has been approved by the FDA for the management of HR+ breast cancer is abemaciclib. Therapy with abemaciclib can enhance human T cell stimulation and may increase the expression of antigen presentation genes in breast cancer cells (Zhang et al., 2021[133]). Subsequent research revealed that monotherapy with abelacilib can boost T cell inflammatory responses and slow the development of tumors. A combination regimen with anti-PDL1 antibody and bemaciclib can promote tumor eradication and immune memory. These findings suggested that both the innate and adaptive immune responses were successfully boosted by combination therapy with abemaciclib and anti-PDL1 antibody. When combined, anti-PDL1 antibody and bemaciclib treatment have shown a lot of promise for use in clinical settings. Zhang et al. looked into the regulating strategy of PDL1 expression and stability because the effectiveness of PDL1 antibody therapy is linked to the protein quantity of PDL1 (Zhang et al., 2018[131]). They discovered that PDL1 regulation involves CDK4. An additional investigation confirmed the exceptional anti-cancer effectiveness of combination treatment using CDK4/6 inhibitors and anti-PDL1 antibody (Deng et al., 2018[29]).

## Conclusion

Maintaining regular cellular activity and delaying the development of cancer depend heavily on cell cycle regulation. This summarizes the significance of cell cycle regulation in tumor. The cell cycle regulates the growth, division, and replication of DNA to allow for the expansion of cells and the production of daughter cells. Many malignancies exhibit dysregulation of the cell cycle, which makes it a desirable target for therapeutic intervention. Drugs that target the cell cycle, such CDK inhibitors, are designed to stop the growth of tumor cells while reestablishing normal cell cycle regulation. It is essential to understand the complexities of cell cycle regulation and how it is dysregulated in malignancy in order to develop therapeutic techniques that work and individualize treatment plans. Scientists may be able to stop cancer cells from growing out of control by focusing on cell cycle regulators and pathways, which will eventually lead to better patient outcomes.

Additional investigation into the regulation of cell cycles has the potential to significantly influence cancer therapies and improve patient outcomes in a number of ways. Further investigation into the control of the cell cycle could uncover new targets for therapy, enabling the creation of focused interventions that interfere with dysregulated cell cycle regulation whilst causing the least amount of damage to normal cells. Further investigation can guide the creation of potent combination therapies that cooperatively target several aspects of the cell cycle apparatus, improving treatment response and getting beyond resistance mechanisms. Extensive research on the regulation of the cell cycle may reveal biomarkers that support patient classification and tailored treatment choices by forecasting patient reactions to specific therapies. Studies can identify drug resistance pathways, which facilitates the creation of countermeasures or preventative measures to improve the long-term effectiveness of cell cycle-targeting treatments. Through identifying the molecular profile of each tumor and customizing treatment plans appropriately, advances in the comprehension of the control of cell cycles can support precision medicine methods. Further investigation could result in the identification and creation of new medicinal products aimed at certain elements or pathways involved in the regulation of the cell cycle, providing enhanced effectiveness and less toxicity.

In summary, additional research into the regulation of the cell cycle in tumor has the potential to transform cancer therapy by enabling the development of tailored, focused therapies, conquering drug resistance, and eventually enhancing patient outcomes. The current state of this field of study will have a significant impact on how cancer therapies are developed in future decades.

## Notes

Ali Hassanzadeh and Navid Shomali contributed equally as first author.

## Declaration

### Ethics approval and consent to participate

Not applicable.

### Availability of data and materials

Not applicable.

### Competing interests

There is no conflict of interest.

### Funding

No funders.

## Figures and Tables

**Table 1:**
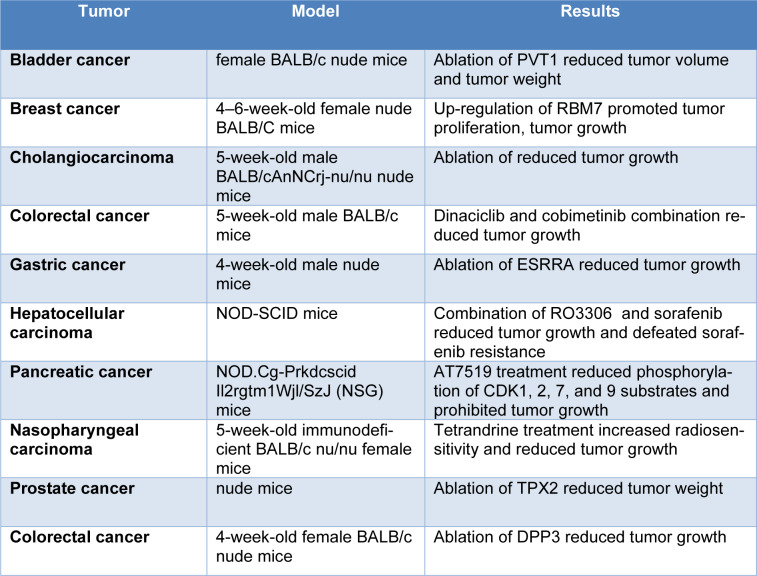
Function of CDK1 in animal models of cancer

**Table 2:**
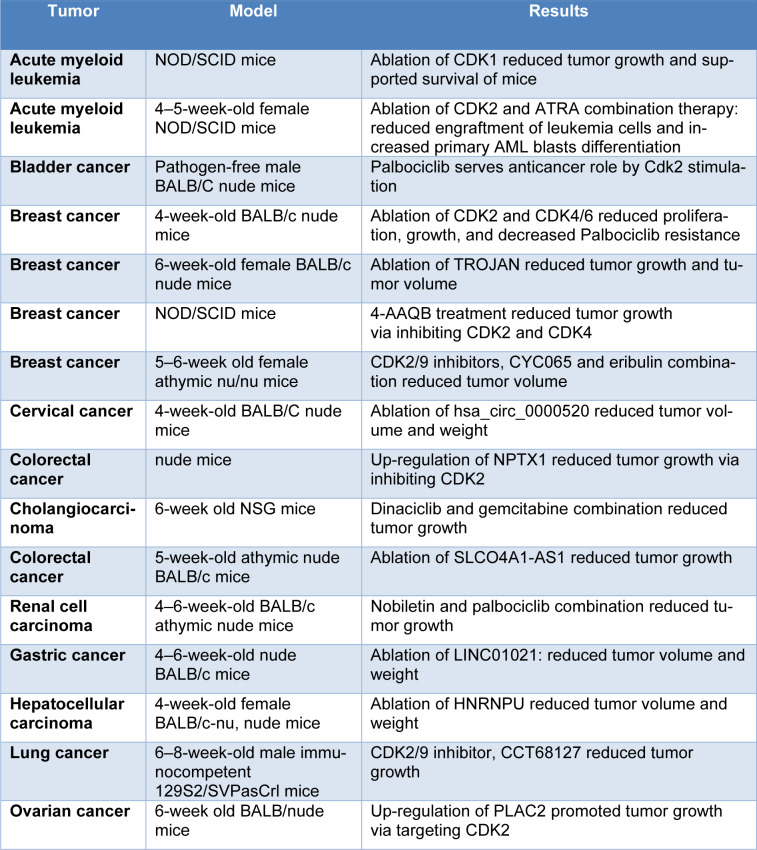
Function of CDK2 in animal models of cancer

**Table 3:**
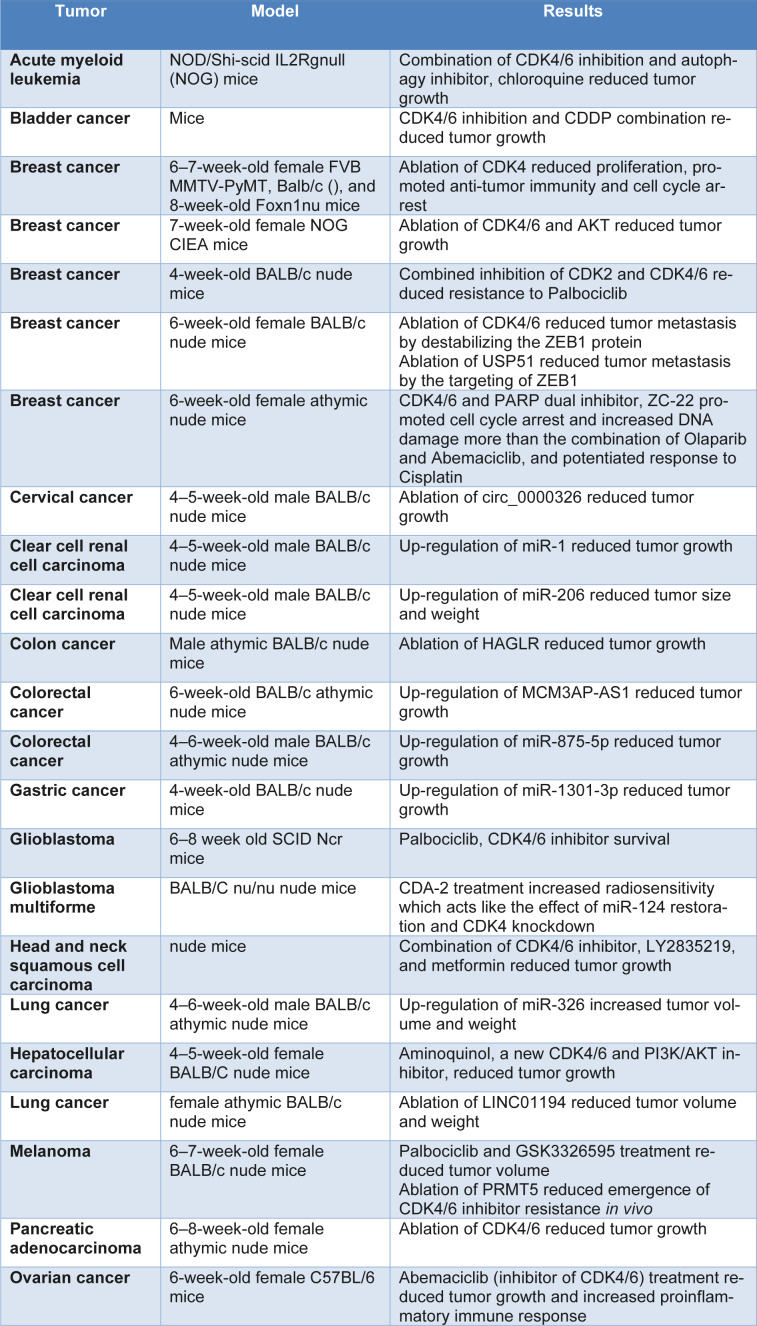
Function of CDK4/6 in animal models of cancer

**Table 4:**
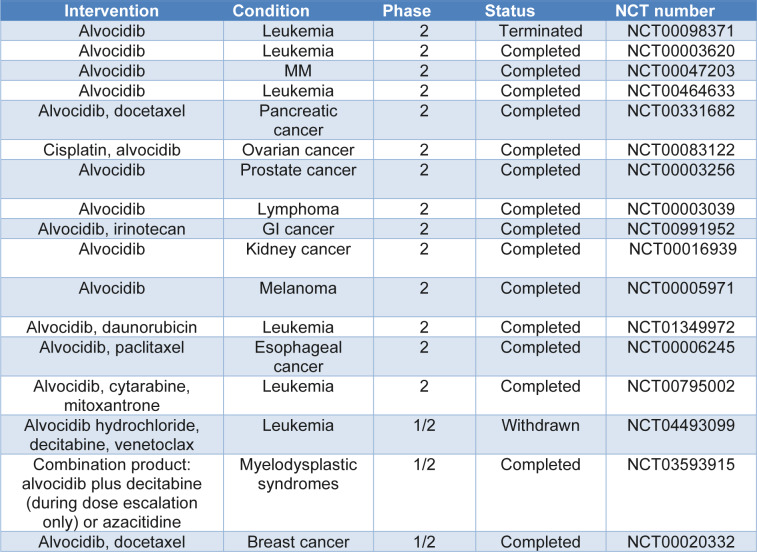
Selected overview of advanced clinical trials of Alvocidib
